# Caring for Oneself or for Others? How Consistent and Inconsistent Profiles of Health-Oriented Leadership Are Related to Follower Strain and Health

**DOI:** 10.3389/fpsyg.2019.02456

**Published:** 2019-11-06

**Authors:** Katharina Klug, Jörg Felfe, Annika Krick

**Affiliations:** Department of Work, Organizational and Economic Psychology, Faculty of Humanities and Social Sciences, Helmut Schmidt University, Hamburg, Germany

**Keywords:** health-oriented leadership, latent profile analysis, employee well-being, self-leadership, inconsistent leadership

## Abstract

Health-oriented leadership consists of three dimensions that contribute to employee health: staff care, i.e., health-specific follower-directed leadership, as well as both leaders’ and followers’ self care, i.e., health-specific self-leadership. This study explores profiles of follower self care, leader self care and staff care, and investigates the relationships with follower health in two samples. We identified four patterns of health-oriented leadership: A consistently positive profile (*high care*), a consistently negative profile (*low care*), and two profiles showing inconsistencies between follower self care, leader self care, and staff care (*leader sacrifice* and *follower sacrifice*). The *high care* profile reported the best health compared to both the *low care* profile and the inconsistent profiles. The *follower sacrifice* profile reported more strain than the *leader sacrifice* profile, while strain and health levels were the least favorable in the *low care* profile. Findings reveal that (in-)consistency between follower-directed leadership and self-leadership contributes to follower strain and health.

## Introduction

Leadership plays a critical role in workplace health promotion: Leaders both represent and shape organizational structures due to their influential role and formal power within the organization (Kelloway and Barling, 2010). Leadership thus represents an important source of workplace demands and resources to influence employee well-being (Bakker and Demerouti, 2016; Nielsen et al., 2017). Accordingly, leaders’ behavior and communication, for example social support, have a direct influence on employee health and well-being (Dormann and Zapf, 1999; Yang et al., 2015). Leadership also affects well-being through a number of indirect pathways: Being responsible for the delegation and organization of tasks, leaders influence their followers’ psychosocial working conditions, such as task variety, autonomy, role clarity, or meaningfulness (Nielsen et al., 2008; Jiménez et al., 2017a). Moreover, leaders themselves experience stressors at work, such as a high workload, multitasking or frequent interruptions (Cavanaugh et al., 2000; Knudsen et al., 2009), and thus affect their followers via crossover of their own strain (Li et al., 2016). Finally, leaders serve as role models showing more or less healthy work behavior which is emulated by followers (Kelloway and Barling, 2010). Overall, a large body of research supports a substantial association between leadership and employee well-being (Kuoppala et al., 2008; Skakon et al., 2010; Montano et al., 2017).

So far, much of the literature in this field has focused on established leadership concepts considered generally effective or ineffective for employee motivation and performance, with leader-member exchange (Graen and Uhl-Bien, 1995) and transformational leadership (Bass and Riggio, 2006) as the most frequently studied constructs (Montano et al., 2017; Nielsen et al., 2017). However, these general concepts were mainly developed to explain performance and thus do not capture specific leadership behaviors and attitudes relevant for employee health and well-being (Vincent-Höper and Stein, 2019). In response, a growing body of literature has emerged to conceptualize domain-specific leadership in order to better understand the links to health and well-being (Gurt et al., 2011; Jiménez et al., 2017b; Vincent-Höper and Stein, 2019).

The concept of health-oriented leadership (Franke et al., 2014) was developed to provide a comprehensive framework for health-relevant leadership attitudes and behavior, accounting for an active role of followers as well as leaders’ role model function. Health-oriented leadership distinguishes between leaders’ and followers’ respective concern for their own health in terms of self-leadership (self care), as well as leaders’ concern for their followers’ health (staff care). The concept has been supported in previous research, as the dimensions of self care and staff care have been shown to contribute to follower strain and health, as well as followers’ working conditions and strain crossover from leaders to followers (Franke et al., 2014; Kranabetter and Niessen, 2017; Horstmann, 2018; Köppe et al., 2018; Santa Maria et al., 2019).

Although clearly correlated, leaders’ and followers’ self care and staff care may not always go hand in hand, and different patterns, including inconsistent behavior (e.g., high staff care, but low self care and vice versa), may occur systematically in subpopulations of leaders and followers. In turn, consistent and inconsistent patterns (i.e., profiles) of self care and staff care likely have differential consequences for health and well-being (see Franke et al., 2014). In line with emerging research on inconsistent leadership (Mullen et al., 2011; Breevaart and Zacher, 2019), we argue that inconsistency between follower-directed leadership and self-leadership creates ambiguity and conflict for followers, ultimately resulting in strain.

Furthermore, identifying patterns of (in-)consistency in health-oriented leadership contributes to a growing body of person-oriented research in occupational health psychology (e.g., Mäkikangas et al., 2013; Bennett et al., 2016; Perko et al., 2016). In leadership research, a few studies have applied person-oriented methods such as profile analysis to identify subtypes of leadership behavior directed to followers such as transformational or paternalistic leadership (Gavan et al., 2009; Chou et al., 2015; Arnold et al., 2017). We aim to expand the conventional understanding of leadership by including leaders’ and followers’ *self*-leadership and shift the focus from effects of isolated variables to persons with similar profiles. Doing so allows to address questions regarding complementary and inconsistent patterns in the interplay between leaders and followers: For example, do leader self care and staff care compensate or compromise each other, and to what extent is their interaction contingent on follower self care? Is a discrepancy between high leader self care and low staff care even more stressful for followers than neither leaders or followers protecting their health? Conversely, could leaders who neglect their own health cause stress for their followers despite practicing good staff care? The incidence and meaning of such profiles have yet to be investigated.

Focusing on follower perceptions of health-oriented leadership (see Kuoppala et al., 2008; Perko et al., 2016), the aim of this study is thus to account for heterogeneity and inconsistency in leadership and health by identifying systematic profiles of follower self care, leader self care and staff care, and to investigate the profiles’ meaning for follower strain and health. We contribute to the literature on leadership and employee health in several ways: First, taking followers’ self-leadership into account, the concept of health-oriented leadership goes beyond a unidirectional perspective of leaders exerting influence on their followers (Franke et al., 2014; see also Wirtz et al., 2017). Second, addressing leaders’ self-leadership theoretically integrates the meaning of role modeling and potential cross-over effects from leaders to followers (see Li et al., 2016; Kranabetter and Niessen, 2017). Third, by investigating the repercussions of inconsistency between follower-directed leadership and self-leadership, we contribute to an emerging literature on the meaning and consequences of inconsistent leadership (Hobman et al., 2009; Mullen et al., 2011; Breevaart and Zacher, 2019). Finally, employees do not experience their leader’s behavior or their own in terms of isolated and unrelated subdimensions. By uncovering constellations of leadership and self-leadership, the person-oriented approach provides a more in-depth understanding of how employees typically experience leadership patterns in relation to health at work.

## Health-Oriented Leadership

Previous research supports the validity and usefulness of domain-specific leadership constructs to predict employee health and well-being. Several studies have shown that health-specific leadership constructs, including health-oriented leadership, explain variance in follower health outcomes above and beyond general task-oriented or transformational leadership (Gurt et al., 2011; Franke et al., 2014; Vincent-Höper and Stein, 2019).

Traditionally, leadership research has been concerned with the behavior of leaders toward followers (Kelloway and Barling, 2010). The concept of health-oriented leadership (HoL) is based on a broader understanding of leadership and further includes both followers’ and leaders’ self*-*leadership (see Manz, 1986; Lovelace et al., 2007). The HoL framework thus consists of three dimensions: *follower self care, leader self care*, and *staff care* (Franke et al., 2014). Whereas staff care represents follower-directed leadership, that is, the extent to which leaders offer resources to promote, or exacerbate demands to risk their employees’ health at work, self care captures both leaders’ and followers’ self-leadership in terms of taking care of their own demands, resources and health at work (Franke et al., 2014).

Follower self care, leader self care and staff care each consist of three components: value of health, health awareness and health behavior. First, value of health describes to the extent to which a person is interested in and gives priority to health issues at work. Second, health awareness describes perceptiveness, that is, the extent to which a person pays attention and reflects upon health issues. In terms of self care, awareness refers to leaders’ and followers’ knowledge of their own signs of strain and conditions which influence their health, while awareness in terms of staff care refers to leaders’ ability to perceive and evaluate strain and well-being among their followers, which can be considered as a precondition for healthy leadership behavior (Turgut et al., 2019). Third, health behavior describes behavioral patterns, activities and habits relevant for maintaining, improving or restoring health (Gochman, 1997; Conner and Norman, 2017). Health behavior can further be differentiated into three facets. First, lifestyle denotes general health-specific habits such as physical exercise and diet. Second, because leadership can be a source of resources but also add to demands (Bakker and Demerouti, 2016; Vincent-Höper and Stein, 2019), work-specific health behavior can be differentiated into a positive facet of health-promoting behavior (e.g., optimizing task organization to reduce demands, supporting OHP participation), and a negative facet of health-risking behavior (e.g., skipping breaks, ignoring exhaustion; Franke et al., 2014; see also Vincent-Höper and Stein, 2019).

Previous research has supported the structure and validity of the HoL framework (Franke et al., 2014; Horstmann, 2018; Santa Maria et al., 2019). Both staff care and follower self care have been shown to contribute to follower health, strain and work-life conflict above and beyond transformational leadership (Franke et al., 2014). Self care has been shown to be susceptible to interventions aimed at increasing personal resources (Krick and Felfe, 2019). Mediation analyses further suggest that reduced staff care explains the crossover of strain from leaders to followers (Köppe et al., 2018) and that part of the positive effect of staff care can be attributed to fostering followers’ self care (Horstmann, 2018; Santa Maria et al., 2019). Leaders’ health awareness has been shown to better explain followers’ task and social resources than abusive leadership (Bregenzer et al., 2019), and to contribute to employee exhaustion in addition to transformational leadership (Kranabetter and Niessen, 2017). The relevance of health awareness and health behavior is further supported by research validating similar concepts, such as health-specific leadership (Gurt et al., 2011), development and health-promoting leadership (Vincent-Höper and Stein, 2019), and health-promoting leadership (Jiménez et al., 2017a). In contrast to these other concepts’ focus on perceived leader behavior, the HoL framework expands established conceptualizations of leadership by including health-specific self-leadership (Vincent-Höper et al., 2017).

## Consistency in Leadership

The dimensions of health-oriented leadership represent interrelated, yet distinct constructs. For example, leaders’ own self care is seen as a favorable precondition for engaging in staff care, because leaders who have strategies to care for themselves may transfer them more easily to their followers. However, the relationships between follower self care, leader self care and staff care tend to be moderate and far from redundancy, leaving considerable room for inconsistency within persons (Franke et al., 2014; Santa Maria et al., 2019).

Although concepts of consistent “good” or “bad” leadership have dominated previous research (Judge and Piccolo, 2004; Schyns and Schilling, 2013; Montano et al., 2017), there is a growing interest in understanding the implications of inconsistency in leadership (e.g., De Cremer, 2003; Mullen et al., 2011). Consistency or inconsistency can mean different things in different contexts: First, consistency may refer to stability in leader behavior across persons, time and situations (De Cremer, 2003). Second, consistency can mean congruence between leaders’ behavior and their own values, or between leaders’ and followers’ values, as reflected in the concept of authentic leadership (Avolio et al., 2004). Finally, inconsistent leadership can refer to the same leader displaying different, seemingly contradictory behaviors, such as transformational leadership and laissez-faire (Mullen et al., 2011; Breevaart and Zacher, 2019) or social support and abusive supervision (Duffy et al., 2002; Hobman et al., 2009). In this study, we focus on (in-)consistency between follower-directed leadership (i.e., staff care) and both leaders’ and followers’ self-leadership (i.e., self care). The common denominator of these different conceptualizations is the notion that inconsistent leader behavior creates uncertainty for followers with detrimental consequences in terms of undermining trust and perceived fairness (e.g., De Cremer, 2003; Breevaart and Zacher, 2019). Accordingly, we argue that due to the leader’s role model position, inconsistency between follower-directed leadership and self-leadership creates ambiguity and conflict for followers as to which health-specific attitudes and behaviors are accepted, rewarded or sanctioned in the workplace, which in turn leads to stress. Furthermore, both stress theory (Lazarus and Folkman, 1984) and leadership research (Schyns and Schilling, 2013) suggest that employees’ perceptions of consistency or inconsistency are more relevant for their experience of stress at work than what leaders may perceive or what might objectively be the case. We thus investigate followers’ individual perceptions of health-oriented leadership.

### Profiles of Health-Oriented Leadership

Franke et al. (2014) alluded to potential inconsistencies between different components of health-oriented leadership: For example, a leader may be aware of strain among his or her followers, but not able or willing to translate this awareness into action. Furthermore, different constellations of self care and staff care may occur, reflecting (in-)consistency between follower-directed leadership and self-leadership. For instance, some leaders may manage to maintain healthy working conditions and encourage self care for both themselves and their followers. For others, a high workload or limited resources may create trade-offs between healthy follower-directed leadership and healthy self-leadership, both of which may require resource investment on the part of leaders and thus be perceived as demanding (see Arnold et al., 2017). Some leaders may react by fostering their followers’ health and self care (i.e., high staff care), but disregarding their own health at work (i.e., low leader self care). While supposedly advantageous for followers, such “self-sacrificing” behavior may be perceived as inconsistent, leading followers to question the authenticity of their leader as a role model (see Gardner et al., 2005; Kelloway and Barling, 2010). In contrast, other leaders may prioritize their own self care at the expense of their followers and show little staff care (i.e., follower “sacrifice”), perhaps due to a perceived trade-off in the utilization of their resources or because these leaders view health as a private matter and do not feel responsible for their followers’ stress at work. Such a pattern likely intensifies demands for followers, limiting their resources for their own self care. Finally, due to limited resources or an organizational climate that values performance over health, leaders may generally show little regard for health at work or may not have the capacity for healthy leadership, neither for themselves nor for their followers. In turn, the consequences of different constellations of leader behavior are likely contingent on followers’ own self care: For example, follower self care may compensate or be diminished by low staff care.

So far, different aspects of health-specific leadership, including leader self care and staff care, have been studied separately and shown independent associations with follower strain (Kranabetter and Niessen, 2017; Horstmann, 2018; Köppe et al., 2018; Santa Maria et al., 2019). However, little is known about heterogeneity in terms of the shape and prevalence of diverse patterns of health-oriented leadership such as those described above. Identifying such groups with specific profiles requires a person-oriented approach. That is, rather than isolating the effects of variables across individuals, a person-oriented analysis aims at identifying subpopulations characterized by meaningful patterns of variables within persons, which cannot be captured with sample-level analyses (Wang et al., 2013; Bergman and Lundh, 2015). Identifying these patterns with methods such as latent profile analysis allows investigating the correlates and consequences of complex variable constellations as employees typically experience them in their work environment in a straightforward manner.

We therefore aim to capture consistency and inconsistency by exploring profiles of health-oriented leadership, characterized by different constellations of follower self care, leader self care and staff care. Given that follower self care, leader self care and staff care are interrelated, we expect to find at least two consistent profiles, that is: high follower self care, high leader self care and high staff care versus a profile characterized by low levels on all three dimensions of health-oriented leadership. However, based on the considerations above, we also expect to find at least two additional profiles, representing groups with inconsistent combinations of low leader self care with high staff care, as well as the opposite pattern. As for follower self care, different constellations are conceivable: For example, when leaders are perceived to protect their followers’ health at the expense of their own (“leader sacrifice”), followers may engage in high self care as they benefit from their leaders’ staff care, but they may also reduce their self care as they follow their leader’s negative example. Conversely, leaders who foster their own health at the expense of their followers (“follower sacrifice”) may limit their team’s resources for self care, but followers may also react with increased self care to compensate for a lack of staff care. It thus remains an open question whether combinations with differing levels of follower self care constitute additional inconsistent profiles. To conclude, we hypothesize the following:

*Hypothesis 1*: Distinct profiles of health-oriented leadership can be identified based on the respective facets of follower self care, leader self care and staff care, including (a) two consistent profiles, characterized by high and low scores on all three dimensions, respectively, and (b) at least two inconsistent profiles, characterized by discrepancies between follower self care, leader self care and staff care.

In order to interpret the profiles in terms of what constitutes “high” or “low” health-oriented leadership and what distinguishes consistent from inconsistent profiles, we differentiate between three facets of self care and staff care, respectively: health awareness, health-promoting behavior and health-risking behavior. Whereas health awareness captures health-relevant attitudes as a precondition of behavior (see Turgut et al., 2019), work-specific health-promoting behavior and health-risking behavior allow to differentiate between (self-)leadership as a source of resources or demands, respectively (see Vincent-Höper and Stein, 2019). Moreover, differentiating between the facets opens up the possibility of detecting potential inconsistencies within the dimensions of self care and staff care, for example between awareness and behavior (see Franke et al., 2014).

### Associations With Follower Strain and Health

Employees’ strain in terms of irritation, psychosomatic complaints and self-rated overall health has been shown to be related to effective leadership in general (Gregersen et al., 2014), as well as to health-oriented leadership specifically (Franke et al., 2014; Köppe et al., 2018). Whereas self-rated health provides a valid and reliable measure of people’s global health status (Singh-Manoux et al., 2006; Baćak and Ólafsdóttir, 2017), irritation and psychosomatic complaints capture cognitive-emotional and physical strain reactions to work stress (Sonnentag and Frese, 2002). We therefore investigated self-rated health, irritation and psychosomatic complaints to assess the relationships of inconsistent health-oriented leadership with both general health and strain-specific mental and somatic symptoms.

Given that profiles of health-oriented leadership represent different constellations of health-relevant resources and risks at work, differential associations with strain and health can be expected. Specifically, we expect employees perceiving consistently high health-oriented leadership to fare better in terms of strain and health than those perceiving consistently low health-oriented leadership, but also better than those perceiving inconsistent patterns. Based on the considerations above regarding inconsistencies, it is plausible to expect that employees with a profile of low leader self care and high staff care (leader “sacrifice”) will be worse off in terms of health than those with a consistently positive profile. Aside from potential leader strain crossing over to followers (Li et al., 2016; Köppe et al., 2018), followers likely experience ambiguity about acceptable or desired health behavior at work and perceive their leader as less authentic (Gardner et al., 2005). This may attenuate positive effects of staff care: Because followers observe the unfavorable health behavior of their leader, they may not fully benefit from staff care and emulate their leader’s low self care (see Cree and Kelloway, 1997; Mullen et al., 2011). Moreover, followers may risk more exhaustion in the long run, as they feel obliged to put in extra effort to help out their overworked leader due to reciprocity, especially if they acknowledge their leaders’ efforts to provide staff care. However, from the perspective of followers, such a pattern should still be less stressful than leaders who show no regard for, or do not have the capacity to consider health at all (i.e., low self care *and* low staff care), or than leaders taking care of their own health, but not for that of their followers.

In contrast, employees with a profile of high leader self care and low staff care (follower “sacrifice”) may get the impression that their leaders save themselves at the expense of their followers who cannot engage in self care and thus be even worse off than those experiencing a consistently negative profile, where at least leaders treat themselves as they treat their followers (see Duffy et al., 2002; Hobman et al., 2009). The discrepancy between leaders’ self care and low staff care in such a pattern likely has negative consequences for their relationship with one another, as followers feel that their leader is not contributing equally to that relationship (Schyns and Wolfram, 2008). In turn, perceptions of injustice may arise and negatively affect follower health (Kivimäki et al., 2007). With regard to differential associations between profiles of health-oriented leadership and follower health, we thus hypothesize the following:

*Hypothesis 2a:* Followers with a consistently positive profile report lower strain and higher health than those with a consistently negative profile.

*Hypothesis 2b:* Followers with a consistently positive profile report lower strain and higher health than those with an inconsistent profile.

*Hypothesis 2c:* Followers with a profile of low leader self care and high staff care (i.e., “leader sacrifice”) report lower strain and better health than those with a profile of high leader self care and low staff care (“follower sacrifice”) or a consistently negative profile.

*Hypothesis 2d:* Followers with a profile of high leader self care and low staff care (“follower sacrifice”) report higher strain and lower health than those with a consistently negative profile.

## Materials and Methods

### Participants and Procedure

We investigated two independent samples of German white-collar employees in the public sector. The samples came from two different administrative organizations, one in finance (tax and revenue offices) and one health insurance. In both organizations, data were collected as part of organizational development projects that focused on occupational health promotion. Afterward, the results were utilized for survey feedback provided by the researchers. Together with members of the organizations’ respective HR departments, the researchers carefully informed participants about the study, about the voluntary nature of participation and confidentiality of the data, and answered participants’ questions about the study. Data were collected between 2016 and 2018. Employees were allowed to complete the online survey during their working time after giving their informed consent to participate.

Sample 1 consisted of *N* = 513 employees in tax and revenue offices. The majority of the sample (74%) were women and the average age was *M* = 45.82 years (*SD* = 8.25). Participants reported having worked with their immediate supervisor between less than one year and 26 years (*M* = 4.08, *SD* = 4.03). Sample 2 consisted of *N* = 776 employees of a regional branch of a health insurance provider. The majority were women (82%) and the average age was *M* = 46.84 years (*SD* = 10.50). Participants reported having worked with their immediate supervisor between less than one year and 27 years (*M* = 5.14, *SD* = 4.83). Both samples consisted of administrative staff.

### Measures

Participants in both samples completed the same measures. Unless stated otherwise, items for each of the following scales were rated on a 5-point Likert scale ranging from 1 = *not at all true* to 5 = *completely true*.

#### Health-Oriented Leadership

The respective awareness and behavior components of self care and staff care were measured with scales from the health-oriented leadership inventory (Pundt and Felfe, 2017). Behavior was further differentiated into the health-promoting and health-risking facets in order to distinguish positive and negative work-specific behaviors. Altogether, nine scales served to assess the three dimensions of follower self care, leader self care and staff care, respectively: health awareness, health-promoting behavior and health-risking behavior. *Follower self care* was measured with 20 items: eight items for *health awareness* (e.g., “I immediately notice when something is wrong with my health”; α = 0.79–82), nine items for *health-promoting behavior* (e.g., “I try to optimize my own work organization in order to reduce my demands (e.g., prioritizing tasks, avoiding disturbances, planning the day)”; α = 0.76–77) and three items for *health-risking behavior* (e.g., “I tend to skip my breaks when there is a lot to do”; α = 0.51–0.58). *Leader self care* was measured with 11 items: three items for *health awareness* (e.g., “My leader takes health-related warning signs seriously”; α = 0.62–0.70), five items for *health-promoting behavior* (e.g., “My leader makes sure to have enough time to relax and recover”; α = 0.77–0.78), and three items for *health-risking behavior* (e.g., “My leader works too much for his/her own good”; α = 0.84–0.91). *Staff care* was measured with 25 items: eight items for *health awareness* (e.g., “My leader immediately notices when something is wrong with my health”; α = 0.87 in both samples), 14 items for *health-promoting behavior* (e.g., “My leader makes sure I have enough time to relax and recover”; α = 0.90–0.93), and three items for *health-risking behavior* (e.g., “It happens often that my leader expects quite a lot of me”; α = 0.57–0.58). The factor structure and criterion validity of the HoL scales have been validated in samples from various sectors, including employees in administration and financial services (Franke et al., 2014; Pundt and Felfe, 2017). Table 1 shows descriptive statistics, internal consistencies, and zero-order correlations for all study variables in both samples. Further supporting construct and criterion validity, inter-correlations between facets from the same HoL dimension (e.g., awareness, promotion, and risk within the dimension of follower self care) tended to be higher than correlations with facets of the respective other dimensions, in addition to substantial correlations with health indicators.

**TABLE 1:** Descriptive statistics and zero-order correlations of all study variables. Correlations for sample 1 are below the diagonal, correlations for sample 2 above.

	**Sample 1**		**Sample 2**		**1**	**2**	**3**	**4**	**5**	**6**	**7**	**8**	**9**	**10**	**11**	**12**	**13**	**14**
	**M (s)**	**α**	**M (s)**	**α**														
**Control variables**																		
1 Gender	−	−	−	−	–	**0.22**	0.02	**0.09**	0.02	0.03	0.05	0.01	0.01	0.01	**–0.13**	0.03	**0.20**	–0.05
2 Age	45.82 (8.25)	−	46.84 (10.50)	−	0.05	–	**0.07**	**0.16**	0.07	0.00	0.00	0.01	–0.07	–0.03	0.03	**0.17**	**0.20**	–**0.23**
**Follower self care**																		
3 Awareness	3.61 (0.70)	0.82	3.73 (0.72)	0.79	0.04	–0.01	–	**0.54**	–**0.30**	**0.20**	**0.17**	–**0.17**	**0.23**	**0.21**	–**0.20**	–**0.36**	–**0.28**	**0.26**
4 Promotion	3.22 (0.66)	0.76	3.22 (0.67)	0.77	0.04	0.02	**0.49**	–	–**0.28**	**0.23**	**0.30**	–**0.21**	**0.30**	**0.36**	–**0.25**	-**0.34**	–**0.23**	**0.27**
5 Risk	3.34 (0.90)	0.58	3.30 (0.88)	0.51	0.03	–0.04	–**0.34**	–**0.31**	–	–**0.09**	–**0.11**	**0.12**	–**0.24**	–**0.22**	**0.39**	**0.32**	**0.21**	–**0.23**
**Leader self care**																		
6 Awareness	3.36 (0.87)	0.70	3.34 (0.86)	0.62	0.00	**0.12**	**0.17**	**0.18**	–0.05	–	**0.65**	–**0.50**	**0.36**	**0.41**	–**0.29**	–**0.16**	–0.07	0.02
7 Promotion	3.37 (0.82)	0.77	3.41 (0.80)	0.78	0.03	**0.11**	**0.12**	**0.23**	–0.04	**0.70**	–	–**0.40**	**0.43**	**0.48**	–**0.29**	–**0.17**	–**0.09**	**0.10**
8 Risk	2.96 (1.08)	0.91	3.09 (0.10)	0.84	0.00	–**0.11**	–**0.13**	–**0.11**	**0.04**	–**0.66**	–**0.55**	–	–0.05	–0.07	**0.19**	**0.24**	**0.13**	–0.05
**Staff care**																		
9 Awareness	3.06 (0.81)	0.87	3.22 (0.85)	0.87	–0.05	–0.01	**0.27**	**0.29**	–**0.19**	**0.24**	**0.33**	–0.05	–	**0.77**	–**0.45**	–**0.21**	–**0.17**	**0.23**
10 Promotion	3.08 (0.82)	0.90	3.36 (0.89)	0.93	–0.03	0.03	**0.15**	**0.24**	–**0.09**	**0.35**	**0.43**	–0.06	**0.62**	–	–**0.46**	–**0.24**	–**0.20**	**0.22**
11 Risk	2.62 (0.92)	0.58	2.43 (0.92)	0.57	–0.08	–0.06	–**0.25**	–**0.29**	**0.44**	–**0.15**	–**0.22**	**0.14**	–**0.36**	–**0.29**	–	**0.24**	**0.10**	–**0.16**
**Health indicators**																		
12 Irritation	2.39 (0.94)	0.87	2.37 (0.95)	0.88	0.02	0.02	–0.43	-**0.40**	**0.39**	–0.04	–0.04	0.04	–**0.16**	–**0.09**	**0.33**	–	**0.56**	–**0.47**
13 Complaints	2.32 (0.88)	0.70	2.45 (0.94)	0.72	**0.13**	0.03	–**0.31**	–**0.29**	**0.31**	–0.08	–**0.10**	0.06	–**0.22**	–**0.11**	**0.27**	**0.53**	–	–**0.56**
14 SRH	3.38 (0.82)	–	3.27 (0.81)	–	–0.05	–0.03	**0.34**	**0.36**	–**0.31**	**0.09**	0.08	–0.07	**0.23**	**0.15**	–**0.27**	–**0.50**	–**0.52**	–

**N = 461–513 for sample 1 (below diagonal), and N = 664–772 for sample 2 (above diagonal) due to casewise deletion of missing values. Significant correlations in boldface (r ≥ 0.04 p < 0.05; r ≥ 0.12 p < 0.01; r ≥ 0.16 p < 0.001). SRH, Self-rated health.**

#### Health Indicators

Cognitive and emotional strain resulting from work was subsumed under the construct of *irritation* as measured with the irritation scale (Mohr et al., 2006). The scale consists of eight items (e.g., “I get grumpy when others approach me”; α = 0.87–0.88). *Psychosomatic complaints* refer to physical symptoms and were measured with an adapted short version of the scale by Mohr (1986) consisting of five items (e.g., “I often suffer from headaches, tensions or back problems”; α = 0.70–0.72). Both scales have been widely used and previously validated among different groups of employees (Müller et al., 2004; Mohr et al., 2006). *Self-rated health* was measured with a single item from the German COPSOQ questionnaire (Nübling et al., 2006), asking participants to rate their current health status from 0 = *worst conceivable health* to 10 = *best conceivable health*. Single items of self-rated health have been supported as valid and reliable measures of people’s general health status (Singh-Manoux et al., 2006), predicting relevant outcomes such as health care expenditures (DeSalvo et al., 2009) and mortality (Idler and Benyamini, 1997). The eleven-point scale was transformed to a five-point scale for the analyses in order to harmonize the response scales for all measures.

#### Control Variables

Because health and psychological distress are influenced by gender and age (Macintyre et al., 1996; Marmot et al., 2012), we controlled for participants’ gender and age in years in the analyses of health indicators. With the exceptions of the health risk facets of follower self care and staff care, respectively, and leader self care awareness in sample 2, all scales showed acceptable or good internal consistencies as indicated by Cronbach’s alpha (see Table 1).

### Statistical Analyses

In order to identify profiles of health-oriented leadership (Hypothesis 1), we conducted a latent profile analyses (LPA), which can be understood as a model-based version of cluster analysis for continuous data (see Zyphur, 2009 for an overview). In LPA, persons are clustered into groups with similar levels and constellations of several variables by modeling a latent categorical variable. Profiles were calculated based on the nine variables measuring health-oriented leadership, that is, the awareness, the health promotion and the health risk facet of each follower self care, leader self care, and staff care. We conducted the analysis using the maximum likelihood procedure in Mplus 6.12 (Muthén and Muthén, 1998–2010). The number of profiles was determined based on the following criteria (Nylund et al., 2007): (1) model fit as indicated by the adjusted Bayesian Information Criterion (aBIC) and likelihood ratio tests (LMRT and VLMRT); (2) classification quality based on entropy and average latent class posterior probabilities (AvePP); (3) profile prevalence (no less than 1% of the sample in one profile), and (4) clarity and theoretical interpretability of the profiles. Lower values for aBIC indicate better model fit. Likelihood ratio tests compare solutions with different numbers of latent profiles: a *p*-value lower than 0.05 suggests that *k* profiles fit the data better than *k*-1 profiles. Entropy illustrates classification accuracy and should be close to 1 (Celeux and Soromenho, 1996). The AvePP evaluates the certainty of assigning an observation to a given profile based on posterior probabilities. Using the most likely latent profile membership, the AvePP was calculated for each profile. For the observations in the most likely profile, an AvePP greater than 0.70 is deemed acceptable (Nagin, 2005).

In order to test for differences between the profiles in irritation, psychosomatic complaints and self-rated health (Hypotheses 2a–d), we calculated ANCOVAs for each variable, controlling for gender and age. We used Bonferroni-adjustments for *post hoc* comparisons between profiles, that is, the conventional significance level of *p* < 0.05 was adjusted by the number of pairwise comparisons.

## Results

As can be seen in Table 1, all facets of health-oriented leadership showed significant small to moderate inter-correlations. In line with the theoretical model, follower self care and staff care were consistently correlated with follower strain and health. Additionally, leader self care partly correlated with follower strain and health: In sample 1, leaders’ health awareness showed positive correlations with follower health and leaders’ health-promoting behavior showed negative correlations with followers’ psychosomatic complaints. In sample 2, all three facets of leaders’ self care were correlated with follower irritation, leaders’ health-promoting and health-risking behavior was correlated with followers’ psychosomatic complaints, and leaders’ health-promoting behavior was correlated with followers’ self-rated health.

### Identifying Profiles

Table 2 shows the LPA results comparing models from two to seven latent profiles. In both samples, we selected the four-profile solution as the final model based on fit criteria, parsimony and interpretability of the profiles. In sample 1, both likelihood ratio tests were marginally significant for four profiles and again for six profiles. We chose the more parsimonious four-profile model, because the six-profile model did not add substantially different and meaningfully interpretable patterns by comparison. In sample 2, four profiles were supported by the likelihood ratio tests. In both samples, the four-profile solution showed good classification quality in terms of entropy, AvePP, and profile proportions.

**TABLE 2:** Latent profile analyses of health-oriented leadership.

**No. of profiles**	**No. of free parameters**	**logL**	**aBIC**	**VLMRT**	**LMRT**	**Entropy**	**AvePP**	**Latent profile counts/proportions**
**Sample 1 (*N* = 513)**								
2	28	–5235.64	10557.13	0.002	0.002	0.74	0.91–0.93	285 (55.6%)/228 (44.4%)
3	38	–5148.23	10412.97	0.248	0.252	0.70	0.82–0.88	137 (26.7%)/197 (38.4%)/179 (34.9%)
**4**	**48**	–**5056**.**86**	**10260**.**90**	**0.081**	**0.084**	**0.73**	**0**.**82**–**0**.**88**	**83 (16.2%)**/**122 >(23.8%)**/**203 (39.6%)**/**105 (20.4%)**
5	58	–4998.94	10175.72	0.246	0.249	0.77	0.84–0.93	137 (26.7%)/185 (36.1%)/69 (13.4%)/29 (5.7%)/93 (18.1%)
6	68	–4954.92	10118.34	0.057	0.058	0.78	0.79–0.89	84 (16.4%)/71 (13.8%)/166 (32.4%)/59 (11.5%)/102 (19.9%)/31 (6.0%)
7	78	–4915.14	10069.44	0.340	0.346	0.77	0.77–0.91	53 (10.3%)/49 (9.6%)/80 (15.6%)/31 (6.0%)/149 (29.1%)/78 (15.2%)/73 (14.2%)
**Sample 2 (*N* = 776)**								
2	28	–7690.40	15478.20	0.000	0.000	0.79	0.92–0.95	472 (60.8%)/304 (39.2%)
3	38	–7519.15	15170.49	0.014	0.014	0.79	0.89–0.93	419 (54.0%)/180 (23.2%)/177 (22.8%)
**4**	**48**	–**7416**.**91**	**15000**.**80**	**0.004**	**0.004**	**0.82**	**0**.**88**–**0**.**93**	**159 (20.5%)**/**161 (20.7%)**/**415 (53.5%)**/**41 (5.3%)**
5	58	–7348.24	14898.24	0.191	0.196	0.80	0.80–0.91	150 (19.3%)/355 (45.8%)/98 (12.6%)/40 (5.2%)/133 (17.1%)
6	68	–7284.80	14806.15	0.204	0.208	0.81	0.84–0.91	128 (16.5%)/107 (13.8%)/36 (4.6%)/124 (16.0%)/342 (44.1%)/39 (5.0%)
7	78	–7235.84	14743.02	0.078	0.081	0.79	0.80–0.89	38 (4.9%)/37 (4.8%)/53 (6.8%)/143 (18.4%)/289 (37.3%)/119 (15.3%)/97 (12.5%)

**Final solution in boldface. logL, Log likelihood; aBIC, sample size-adjusted Bayesian Information Criterion; VLMRT, Vuong-Lo-Mendell likelihood ratio test; LMRT, Lo-Mendell likelihood ratio test; AvePP, average latent class posterior probabilities.**

**FIGURE 1:**
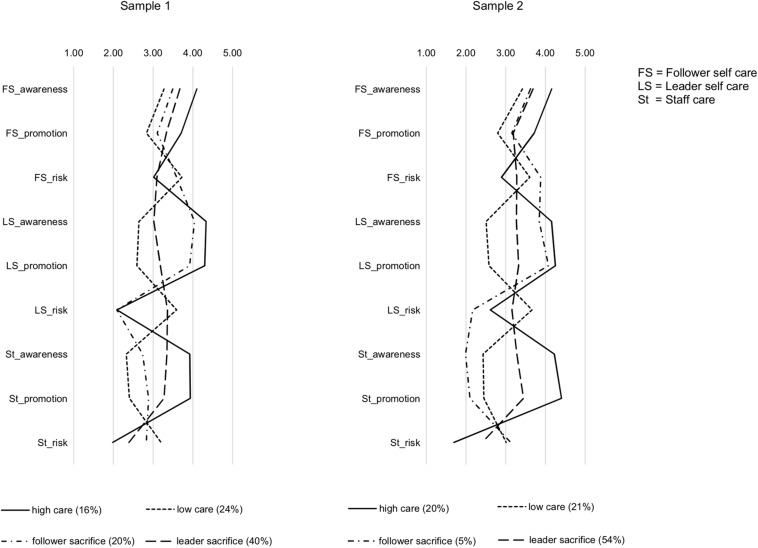
Profiles of health-oriented leadership: Mean scores of follower self care variables (awareness, promotion, risk), leader self care variables (awareness, promotion, risk) and staff care variables (awareness, promotion, risk) across the four profiles.

Figure 1 illustrates the four profiles in terms of mean scores for all facets of health-oriented leadership. The first profile was labeled *high care* (16% of sample 1 participants and 20% of sample 2 participants), because it was characterized by high follower self care (i.e., high awareness, high health-promoting behavior and low health-risking behavior), high leader self care and high staff care. The leader self care dimension was slightly more pronounced in sample 1 and the staff care dimension more pronounced in sample 2, but the profile shape was highly similar in both samples. The second profile was labeled *low care* (24% in sample 1 and 21% in sample 2) because it showed the opposite pattern, that is, low follower self care, low leader self care and low staff care. The third profile was labeled *follower sacrifice* (20% in sample 1, and 5% in sample 2), because it was characterized by high leader self care but low staff care, with moderate levels of follower health awareness and health-promoting behavior and high levels of follower health-risking behavior. The profile was more pronounced in sample 2, as can be seen in lower scores on health awareness and health-promoting behavior in the staff care dimension, but the discrepancy between leader self care and staff care is clearly visible in both samples. Finally, the fourth profile showed moderate levels on most variables. Because in this profile, leaders’ own health-risking behavior was higher than their health-risking behavior toward followers, we named this profile *leader sacrifice* (40% in sample 1 and 54% in sample 2). In summary, we identified two profiles representing consistently high and low health-oriented leadership, as well as two inconsistent profiles characterized by opposite discrepancies between leader self care and staff care, all of which were replicated across two independent samples. Hypotheses 1a and 1b were thus supported.

### Differences in Strain and Health Across the Profiles

The ANCOVA results depicted in Table 3 show that after adjusting for gender and age, the four profiles differed significantly in all three health indicators. First, we expected better health in the *high care* (hc) profile than in the *low care* (lc) profile (Hypothesis 2a). Bonferroni-adjusted *post hoc* tests (i.e., adjusted for eight pairwise comparisons between the four profiles) indicated that participants with the *high care* profile reported lower irritation (M_hc_ – M_lc_ = −0.74, *SE* = 0.13, *p* < 0.001), fewer psychosomatic complaints (M_hc_ – M_lc_ = −0.68, *SE* = 0.12, *p* < 0.001) and better health (M_hc_ – M_lc_ = 0.79, *SE* = 0.11, *p* < 0.001) in sample 1. The same pattern emerged in sample 2 (M_hc_ – M_lc_ = −0.83, *SE* = 0.11, *p* < 0.001 for irritation; M_hc_ – M_lc_ = –0.58, *SE* = 0.10, *p* < 0.001 for psychosomatic complaints; M_hc_ – M_lc_ = 0.54, *SE* = 0.09, *p* < 0.001 for self-rated health). Hypothesis 2a was supported.

**TABLE 3:** Descriptive statistics and ANCOVA of health indicators across profiles, controlling for gender and age.

**Profiles**	**Irritation**				**Psychosomatic complaints**				**Self-rated health**			
	**M (SE)**	***F (df)***	***p***	**η^2^**	**M (SE)**	***F (df)***	***p***	**η^2^**	**M (SE)**	***F (df)***	***p***	**η^2^**
Sample 1												
P1 High care	1.95 (0.11)				1.88 (0.10)				3.81 (0.09)			
P2 Low care	2.68 (0.09)				2.55 (0.08)				3.03 (0.08)			
P3 Leader sacrifice	2.29 (0.07)				2.14 (0.06)				3.52 (0.06)			
P4 Follower sacrifice	2.55 (0.09)				2.41 (0.09)				3.26 (0.08)			
		12.62(3,499)	0.000	0.07		12.83(3,499)	0.000	0.07		11.77(3,497)	0.000	0.10
Sample 2												
P1 High care	1.94 (0.08)				1.98 (0.08)				3.55 (0.07)			
P2 Low care	2.77 (0.08)				2.56 (0.08)				3.02 (0.07)			
P3 Leader sacrifice	2.37 (0.05)				2.34 (0.05)				3.30 (0.05)			
P4 Follower sacrifice	2.53 (0.15)				2.56 (0.15)				2.92 (0.13)			
		21.35(3,710)	0.000	0.08		11.84(3,709)	0.000	0.05		14.40(3,698)	0.000	0.06

*N = 505 for irritation and for psychosomatic complaints, and N = 503 for self-rated health in sample 1; N = 716 for irritation, N = 715 for psychosomatic complaints, and N = 705 for self-rated health due to listwise exclusion of missing values. P = Profile.*

Second, we expected better health in the *high care* profile than in the two inconsistent profiles, that is, the *follower sacrifice* (fs) and the *leader sacrifice* (ls) profiles, respectively (Hypothesis 2b). *Post hoc* tests indicated that the *high care* profile showed lower irritation than the *follower sacrifice* profile (M_hc_ – M_fs_ = −0.51, *SE* = 0.13, *p* < 0.001), as well as fewer psychosomatic complaints (M_hc_ – M_fs_ = −0.54, *SE* = 0.13, *p* < 0.001) and better health (M_hc_ – M_fs_ = 0.55, *SE* = 0.12, *p* < 0.001) in sample 1, and also in sample 2 (M_hc_ – M_fs_ = −0.58, *SE* = 0.17, *p* = 0.002 for irritation; M_hc_ – M_fs_ = −0.58, *SE* = 0.16, *p* = 0.002 for psychosomatic complaints; M_hc_ – M_fs_ = 0.63, *SE* = 0.14, *p* < 0.001 for self-rated health). Participants with the *high care* profile also reported lower irritation than the *leader sacrifice* profile (M_hc_ – M_ls_ = −0.34, *SE* = 0.12, *p* = 0.027), as well as better health (M_hc_ – M_ls_ = 0.29, *SE* = 0.10, *p* = 0.034) in sample 1. The same results were found in sample 2, in addition to fewer psychosomatic complaints in the *high care* profile (M_hc_ – M_ls_ = −0.42, *SE* = 0.09, *p* < 0.001 for irritation; M_hc_ – M_ls_ = −0.36, *SE* = 0.09, *p* < 0.001 for psychosomatic complaints; M_hc_ – M_ls_ = 0.25, *SE* = 0.08, *p* = 0.007 for self-rated health). Hypothesis 2b was thus largely supported.

Third, we expected better health in the *leader sacrifice* profile compared to both the *follower sacrifice* profile and to the *low care* profile (Hypothesis 2c). The *leader sacrifice* profile reported marginally lower irritation (M_ls_ – M_fs_ = −0.27, *SE* = 0.11, *p* = 0.094), marginally fewer psychosomatic complaints (M_ls_ – M_fs_ = −0.27, *SE* = 0.10, *p* = 0.054) and better health (M_ls_ – M_fs_ = 0.50, *SE* = 0.09, *p* < 0.001) than the *follower sacrifice* profile in sample 1. In sample 2, the leader sacrifice and follower sacrifice profile differed only in terms of health (M_ls_ – M_fs_ = 0.38, *SE* = 0.14, *p* = 0.028). Compared to the *low care* profile, the *leader sacrifice* profile showed lower irritation (M_ls_ – M_lc_ = −0.40, *SE* = 0.11, *p* = 0.001), fewer psychosomatic complaints (M_ls_ – M_lc_ = −0.41, *SE* = 0.10, *p* < 0.001) and better health (M_ls_ - M_lc_ = 0.50, *SE* = 0.09, *p* < 0.001) in sample 1. The same pattern emerged in sample 2, though the difference was only marginally significant at *p* < 0.10 for psychosomatic complaints (M_ls_ – M_lc_ = −0.41, *SE* = 0.09, *p* < 0.001 for irritation; M_ls_ – M_lc_ = −0.22, *SE* = 0.09, *p* = 0.066 for psychosomatic complaints; M_ls_ – M_lc_ = 0.29, *SE* = 0.08, *p* = 0.001 for self-rated health). Hypothesis 2c was thus largely supported in terms of the difference between *leader sacrifice* and *low care*, but only partially with regard to differences between *leader sacrifice* and *follower sacrifice.*

Fourth, we expected poorer health for the *follower sacrifice* profile compared to the *low care* profile (Hypothesis 2d). However, there were no significant differences on any of the health indicators between these two profiles. Hypothesis 2d was not supported.

**FIGURE 2:**
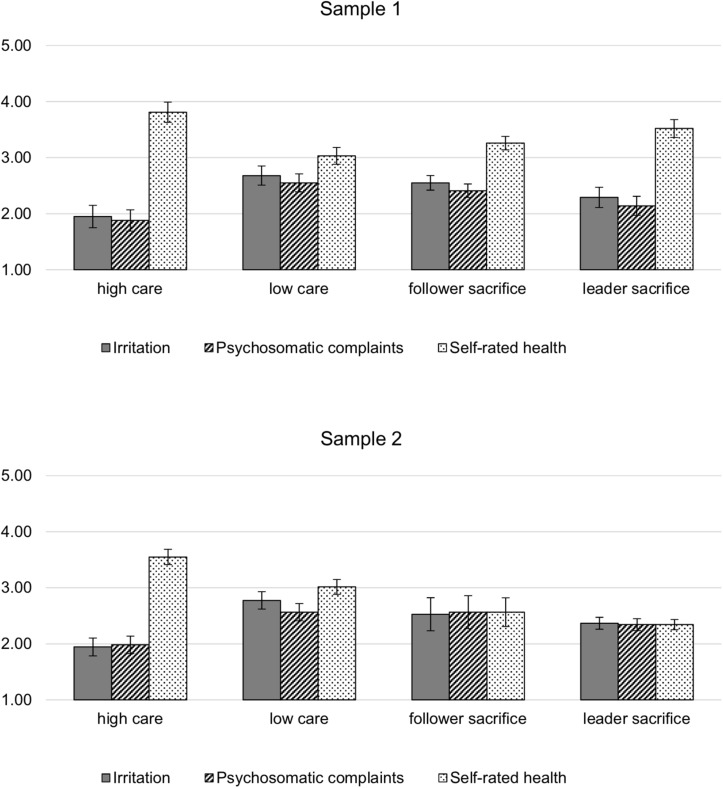
Mean scores and 95%-confidence intervals of irritation, psychosomatic complaints and self-rated health across the four profiles of health-oriented leadership.

Figure 2 summarizes the mean scores on all health indicators across the profiles. To conclude, with the exception of Hypothesis 2d, our expectations regarding health differences between the profiles were by and large supported: Significant differences in strain and health emerged across the profiles in both samples, with the *high care* profile showing the most favorable levels, followed by the *leader sacrifice* profile, whereas *follower sacrifice* and *low care* showed the most unfavorable levels and did not systematically differ from one another.

## Discussion

The aim of this study was to account for heterogeneity among employees in their experience of leadership, strain and health, and to investigate the implications of consistent and inconsistent leadership in a person-oriented analysis. We present a novel approach to examine health-oriented leadership by identifying profiles representing different constellations of follower self care, leader self care and staff care, and also account for both leaders’ and followers’ self-leadership. Our findings replicate and extend previous research on the validity of health-oriented leadership (Franke et al., 2014; Horstmann, 2018; Santa Maria et al., 2019), and contribute to the discussion about the nature and consequences of inconsistency in leadership (Duffy et al., 2002; De Cremer, 2003).

### Profiles of Health-Oriented Leadership and Follower Health

In line with our expectations, we identified four distinct profiles of health-oriented leadership. These profiles were strikingly similar in both samples. First, the *high care* profile (16% in sample 1; 20% in sample 2) was characterized by relatively high scores on all positive facets of follower self care, leader self care and staff care, and low scores on health-risking behavior, respectively. Second, the *low care* profile (24% in sample 1; 21% in sample 2) showed the reverse pattern of low scores on all positive facets and high scores on health-risking behavior. Third, the *follower sacrifice* profile (20% in sample 1 and 5% in sample 2) was characterized by a discrepancy between high leader self care and low staff care, as well as low follower self care, particularly in terms of high health-risking behavior. The inconsistency between follower-directed leadership and self-leadership was more pronounced in the second sample as indicated by higher risk in the follower self care dimension and overall lower staff care (i.e., low health awareness, low health promotion and high risk) compared to sample 1. Fourth, the *leader sacrifice* profile was characterized by overall moderate scores on all variables, but a small discrepancy between follower-directed leadership and self-leadership in terms of health-risking behavior: leaders were perceived to show more health-risking behavior toward themselves than toward their followers who, in turn, did not risk their health much. This pattern was clearer in the first sample, whereas followers’ own health-risking behavior was moderate in sample 2. With a prevalence of 40% (sample 1) to 54% (sample 2), this was the most common profile in both samples.

The first two profiles (i.e., *high care* and *low care*) represent consistency in terms of high or low health-oriented leadership for both followers and leaders, supporting Hypothesis 1a. In support of Hypothesis 1b, the third and fourth profile (i.e., *follower sacrifice* and *leader sacrifice*) show that inconsistency in terms of discrepancies between follower-directed leadership and self-leadership occurs as well. With regard to follower self care, the two inconsistent profiles were similar in terms of health awareness and health-promoting behavior, but health-risking behavior was clearly higher in the *follower sacrifice* profile. The discrepancy with low health-risking behavior on the part of leaders in combination with relatively high risk on the staff care dimension renders employees with a *follower sacrifice* profile particularly vulnerable as they seem to have few resources to protect their health at work. Regarding the open question how follower self care combines with different inconsistent profiles, both the *follower sacrifice* and the *leader sacrifice* profile showed relatively low follower self care compared to the consistently positive *high care* profile (except for risk in sample 1). It thus appears that discrepancies between leader self care and staff care limit followers’ resources for self care rather than prompting compensatory efforts to increase healthy self-leadership. Within the dimensions of self care and staff care, the different facets tended to be consistent, that is, where health awareness and health-promoting behavior were higher, health-risking behavior was lower and vice versa. Taken together, the findings show that employees do not only experience health-oriented leadership as generally high or low, but as part of consistent and inconsistent patterns: Facets of self care and staff care are perceived in the context of complex patterns with the respective other facets, which has important implications for employee strain and health.

Our expectations regarding differences in irritation, psychosomatic complaints and health between the four profiles were mostly supported as well. First, participants with the *high care* profile reported the most favorable health, showing lower strain and better health than participants with the *low care profile* (Hypothesis 2a), but more importantly, also in comparison to the two inconsistent profiles of *follower sacrifice* and *leader sacrifice* (Hypothesis 2b). Thus, it appears that low leader self care may diminish the benefits of staff care, either because the leader as a role model is perceived as less authentic (Gardner et al., 2005; Kranabetter and Niessen, 2017), due to crossover of strain (Ten Brummelhuis et al., 2014; Köppe et al., 2018), or because followers feel the need to neglect their own self care to help out their leader. Indeed, the *leader sacrifice* profile showed mostly lower follower self care than the consistently positive *high care* profile.

The expectation that a *leader sacrifice* profile, though not consistently positive, would still be more favorable from a follower perspective than *low care* or *follower sacrifice* (Hypothesis 2c), was largely supported with regard to differences between *leader sacrifice* and *low care.* However, differences between the *leader sacrifice* profile and the *follower sacrifice* were marginal and less consistent, perhaps because the *leader sacrifice* profile was not as strongly pronounced as we expected. In comparison to the other profiles, the mean scores on almost all facets of health-oriented leadership were on moderate levels. However, the pattern of leaders showing more health-risking behavior toward themselves than toward their followers justifies the label *leader sacrifice.*

Contrary to our expectations, there were no health differences between the *follower sacrifice* profile and the *low care* profile (Hypothesis 2d) in either sample. Thus, it does not appear that employees in the *follower sacrifice* profile perceive their situation as particularly unfair compared to consistently “unhealthy” leadership. Though we can only speculate about the dynamics underlying the different profiles, several explanations are conceivable: First, from a follower perspective, it may simply be that leader self care is not as salient for follower strain as staff care or their own self care. Second, followers may attribute the discrepancy between leader self care versus their own low self care and low staff care to external circumstances rather than their leader, and thus not perceive unfair treatment. Conversely, employees with a *low care* profile may just as well attribute their situation to their leader’s disregard for their health, with negative consequences for the quality of the relationship or perceptions of fairness (see Kivimäki et al., 2007; Schyns and Wolfram, 2008).

### Theoretical and Practical Implications

Taken together, our findings support the concept of health-oriented leadership (Franke et al., 2014), as they underline the value of considering healthy leadership in the context of both leaders’ and followers’ self*-*leadership (Kranabetter and Niessen, 2017; Horstmann, 2018). Bivariate correlations between the components of health-oriented leadership, strain and health are in line with previous findings (Franke et al., 2014; Santa Maria et al., 2019) and extend existing validity. Moreover, profiles of health-oriented leadership and their differential associations with follower strain show the importance of considering consistency in leadership.

Our findings extend existing research on inconsistency in leader behavior across time and situations (De Cremer, 2003) or in terms of seemingly opposing leader behaviors (Duffy et al., 2002; Hobman et al., 2009; Mullen et al., 2011) by considering consistency between follower-directed leadership and self-leadership. In contrast to previous studies (Duffy et al., 2002; Hobman et al., 2009) reporting the least favorable follower outcomes under conditions of high supervisor support *and* high undermining or abusive behavior, our findings suggest that consistency as such is not always better than inconsistency, at least not in comparison to consistently unhealthy (self-)leadership. Moreover, inconsistency can also occur to the followers’ advantage, as in the *leader sacrifice* profile. In order to better understand how and why different patterns of inconsistency affect strain and health at work, further theory development may take into account the underlying mechanisms, such as follower attributions (Martinko et al., 2007).

With regard to practical implications, our findings suggest that leaders should be aware of their role model position and impact on follower health. Consequently, occupational health promotion initiatives should target both employees and supervisors. Moreover, leaders may be supported in assuming responsibility for health at work and trained in providing healthy working conditions for their employees, but also for themselves (see Kranabetter and Niessen, 2017). Especially with regard to inconsistencies between healthy leadership and self-leadership, interventions focusing on the individual (e.g., training or coaching) could tackle leaders’ experiences, beliefs and attributions regarding conflicts and trade-offs between caring for themselves or for their followers’ well-being. Interventions aimed at the organizational environment may focus on work (re-)design to ensure leaders have adequate resources and reasonable demands to accomplish both high self care *and* high staff care, in addition to creating favorable conditions for employees’ self care.

### Strengths, Limitations, and Recommendations for Future Research

This study has some limitations that should be kept in mind when interpreting the results. First, due to the cross-sectional design we cannot infer causal relationships between health-oriented leadership and follower strain. It is conceivable that strained individuals rate their leader, as well as their own health behavior less positively. However, reverse causation would not explain the emergence of different inconsistent profiles in terms of *leader sacrifice* and *follower sacrifice.* Moreover, in line with the person-oriented approach (Bergman and Lundh, 2015), our goal with this study was to identify meaningful patterns of health-oriented leadership and investigate their respective relationships with follower strain rather than isolating causal effects of single variables. Related to this issue, there is no consensus about minimum sample sizes for LPA, and we were not able to calculate the required sample size to detect the “true” number of profiles *a priori*. LPA is an exploratory procedure and the required sample size to detect profiles depends on various factors such as the number, size and structure of profiles or the degree of separation between them (Nylund et al., 2007; Park and Yu, 2018). Though we hypothesized at least four profiles, the exact number and structure were impossible to derive from the literature, because our study was the first to investigate HoL profiles. Hence, the number of groups in the ANCOVAs could not be predicted either. However, we were able to replicate the number and structure of the four profiles in two independent samples, which is a considerable strength of this study and underlines its validity. Future research may not only replicate the findings and address reciprocal relationships between profiles of health-oriented leadership and strain, but also analyze stability and change in the profiles themselves over time via latent transition analysis or trajectory analysis (see Perko et al., 2016).

Second, we cannot rule out that relationships between leadership and strain were inflated by common method bias (Podsakoff et al., 2003), as both were rated by followers. Yet again, common method bias could not plausibly explain the emergence and differential implications of the two inconsistent profiles – instead the contrary in terms of homogeneous groups should be expected in the presence of common method bias. Moreover, it is common practice and conceptually sensible to focus on follower perceptions when investigating individual consequences of leadership, because “leader behavior can only have an effect when it is perceived by followers” (Schyns and Schilling, 2013, p. 140). Nevertheless, future research would benefit from incorporating the leaders’ perspective for triangulation purposes, as well as leaders’ own strain and health to better understand the reciprocal dynamics between leaders and their followers that bring about different profiles: It is for example conceivable that the profiles we identified affect the leaders’ health as well (see Arnold et al., 2017; Wirtz et al., 2017). Furthermore, followers’ strain and behavior may influence a leaders’ practice of health-oriented leadership.

Third, our study design did not include other work or organizational characteristics that may explain the mechanisms between the profiles and health indicators, such as demands, control, organizational support, or fairness perceptions (Bregenzer et al., 2019; Turgut et al., 2019), nor employee characteristics which may influence the perception of leadership such as job type or tenure. On the other hand, a strength of this study was that we considered a range of different facets and provided a detailed account of health-oriented leadership, including not just behavior but also health awareness, and not just follower-directed leadership but also self-leadership. As this study constitutes the first step of identifying different profiles of health-oriented leadership, further studies may include other variables and focus on explaining and predicting the occurrence of different profiles. Such research may provide valuable insights for practice by addressing the questions under which conditions leaders and followers manage to engage in consistently positive health-oriented leadership.

## Conclusion

This study is the first to investigate profiles of health-relevant leadership constructs and thus to account for subpopulations with different leadership experiences, particularly in terms of consistency between follower-directed leadership and self-leadership. We identified four profiles representing meaningful constellations of follower self care, leader self care and staff care, and showing differential relationships with follower strain and health. The *high care* profile was most favorable with regard to health, followed by the *leader sacrifice* profile, whereas both the *follower sacrifice* profile and the *low care* profile showed the least favorable health. The results suggest that in order to prevent employee strain, organizations should aim at improving not only follower-directed leadership, but also foster healthy self-leadership among both leaders and followers.

## Data Availability Statement

The raw data supporting the conclusions of this manuscript will be made available by the authors, without undue reservation, to any qualified researcher.

## Ethics Statement

Ethical review and approval was not required for the study on human participants in accordance with the local legislation and institutional requirements. The patients/participants provided their written informed consent to participate in this study.

## Author Contributions

KK and JF developed the research question and study design. JF collected the data. KK performed the statistical analysis and wrote the first draft of the manuscript, which JF and AK supported and checked. All authors contributed to revising the manuscript, and read and approved the submitted version.

## Conflict of Interest

The authors declare that the research was conducted in the absence of any commercial or financial relationships that could be construed as a potential conflict of interest.
